# Association between serum carotenoids levels and endometriosis risk: evidence from the National Health and Nutrition Examination Survey

**DOI:** 10.3389/fnut.2025.1513191

**Published:** 2025-02-04

**Authors:** Jian Huang

**Affiliations:** Clinical Laboratory Center, The First Affiliated Hospital of Guangxi Medical University, Nanning, China

**Keywords:** endometriosis, risk, carotenoids, serum lutein/zeaxanthin levels, NHANES

## Abstract

**Background:**

The relationship between serum levels of carotenoids and endometriosis remains largely unknown. The aim of this study is to assess the association between serum levels of major carotenoids (*α*-carotene, *β*-carotene, β-cryptoxanthin, lutein/zeaxanthin, and trans-lycopene) and the risk of endometriosis in US women.

**Methods:**

The data were obtained from the 2001–2006 National Health and Nutrition Examination Surveys (NHANES), which included a total of 3,636 women aged 20 to 54. Serum levels of *α*-carotene, *β*-carotene, β-cryptoxanthin, lutein/zeaxanthin, and trans-lycopene were measured using high performance liquid chromatography (HPLC) with photodiode array detection. Endometriosis was defined as self-report. Weighted multivariate logistic regression analyses were conducted to evaluate the associations of the serum levels of the major carotenoids with endometriosis risk. Additionally, restricted cubic spline (RCS) was employed to assess the possibility of nonlinear associations. Finally, subgroup analyses were utilized to estimate the influence of several covariates on the associations.

**Results:**

Weighted multivariate logistic regression analyses showed that, after adjusting for all covariates taken into account, there was a significant association between serum lutein/zeaxanthin levels and reduced risk of endometriosis (Quartile 3 vs. Quartile 1: odds ratio [OR] = 0.62, 95% confidence interval [CI]: 0.42–0.90; Quartile 4 vs. Quartile 1: OR = 0.54, 95% CI: 0.36–0.81, *P* for trend = 0.001). However, no significant associations of serum levels of other carotenoids with endometriosis were found in multivariable-adjusted models that included all covariates. RCS analysis did not reveal any non-linear relationships. Subgroup analyses indicated that the inverse association between serum lutein/zeaxanthin levels and reduced endometriosis risk was significant only in individuals under 40 years of age, in both White and non-White populations, in smokers, and among those who had ever used oral contraceptives.

**Conclusion:**

Serum lutein/zeaxanthin levels may offer protective effects against endometriosis in specific subpopulations. Further prospective research is necessary to validate these findings.

## Introduction

Endometriosis is a chronic inflammatory condition characterized by the implantation and growth of endometrial-like tissue outside the uterine cavity, usually in the pelvis. It’s estimated that endometriosis affects between 2 and 10% of women in the general population (1). Endometriosis most frequently presents with symptoms such as chronic pelvic pain, pain during or after sex, excessive bleeding, and infertility (1). Other associated symptoms include constipation, bloating, diarrhea, fatigue, depression, anxiety, and problems with the urinary system (2). The effects of endometriosis on physical health, emotional stability, and productivity are profound, causing a considerable strain on patients’ quality of life, as well as economic and social hardships for families and society (3). At present, no curative treatments are available. While the exact aetiology of endometriosis remains largely unknown, neurological, immunological, and hormoral factors are considered important contributors to the pathogenesis and development of endometriosis (1). In addition, evidence from clinical studies has suggested that nutritional factors including vitamin D, lipids, and gut microbiota may be implicated in the risk of developing endometriosis (4, 5).

Carotenoids are a class of bioactive red, orange, and yellow compounds produced by a variety of plants, fungi, and bacteria. More than 1,100 naturally occurring carotenoids have been identified, with around 50 being included in human diets and capable of being absorbed and processed by the body (6). Despite the variety, six carotenoids, specifically *β*-carotene, β-cryptoxanthin, ɑ-carotene, lycopene, lutein, and zeaxanthin, comprise more than 95% of the carotenoids present in the bloodstream (7). Carotenoids play important roles in biological function, including antioxidant, anti-inflammatory, and anti-carcinogenic activity (8). Numerous studies have suggested a strong inverse association between carotenoid levels and the risk of type 2 diabetes, stroke, coronary heart disease, asthma, and certain cancers, including lung and breast cancer (9). In addition, several studies have been undertaken to evaluate the association between carotenoids and endometriosis risk. Some studies have suggested a positive relationship. For instance, Trabert and colleagues found a suggestion of enhanced risk of endometriosis with increased *β*-carotene consumption (fourth quartile vs. lowest: odds ratio [OR] 1.6, 95% confidence interval [CI]: 1.0–2.5) (10). However, using data from The Nurses’ Health Study II (NHS II), Harris et al. (11) revealed that only intake of *β*-cryptoxanthin but not other cateroids (ɑ-carotene, β-carotene, lutein, zeaxanthin and lycopene) was associated with reduced risk of endometriosis. The findings from current studies are still inconclusive; further research is necessary, particularly studies examining the association between circulating carotenes levels and the risk of endometriosis.

Using national survey data, we evaluated the associations between serum levels of major carotenoids (ɑ-carotene, *β*-carotene, β-cryptoxanthin, lutein/zeaxanthin, and trans-lycopene) and the risk of endometriosis in a representative sample of U.S. women. We also sought to investigate whether several factors such as smoking status and oral contraceptive use would influence the associations.

## Materials and methods

### Study design

**Figure 1:**
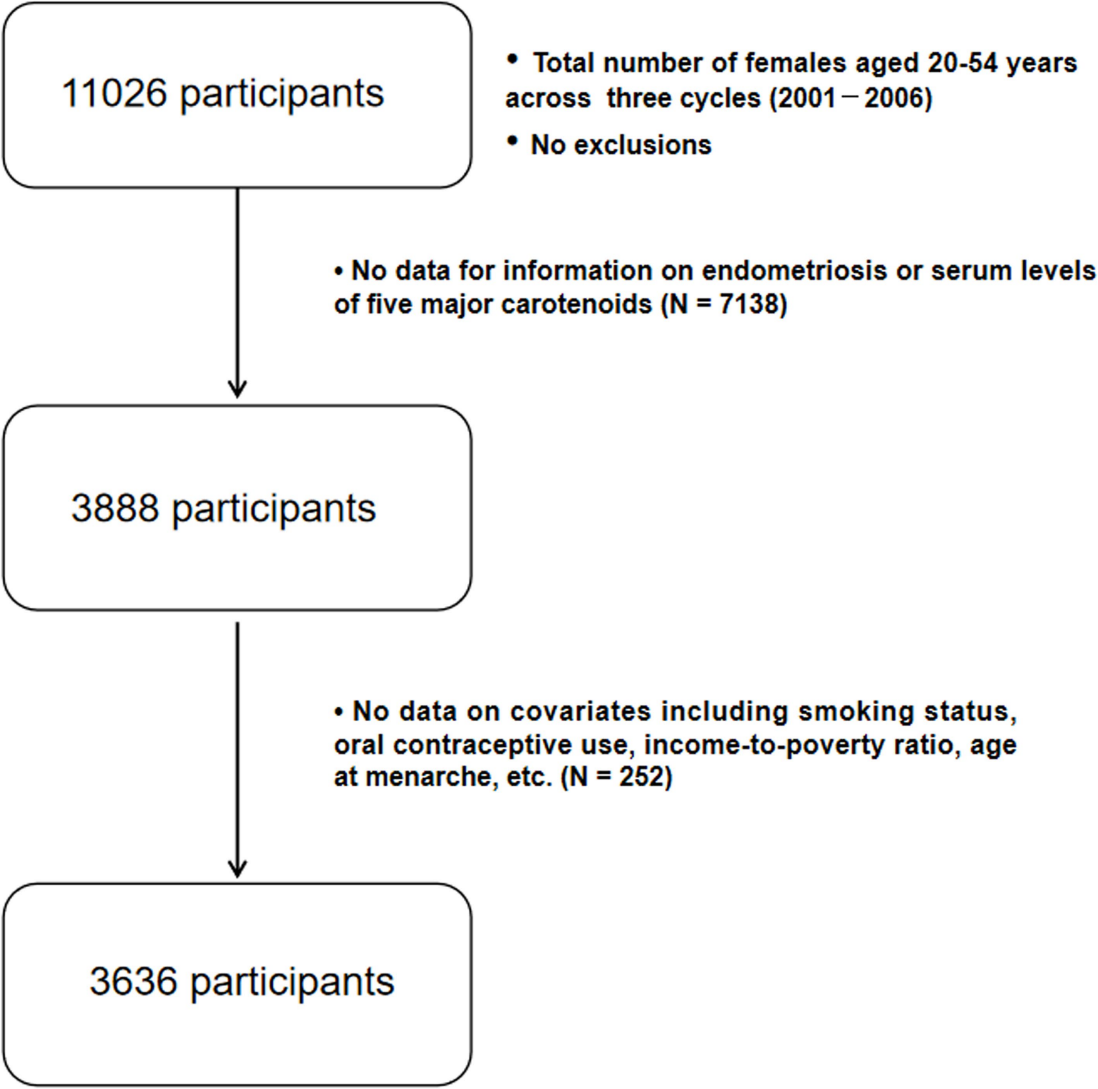
Flow chart for study participants.

We conducted a cross-sectional study using data from the National Health and Nutrition Examination Survey (NHANES) conducted by the Centers for Disease Control and Prevention for the years 2001–2002, 2003–2004 and 2005–2006. The analysis is restricted to these years as endometriosis and serum carotenoids information is available exclusively for this period. NHANES is a continuous and annual survey which evaluates a representative sample of non-institutionalised US civilians (12). Participants are selected by a complex, multistage probability design. The data collection process involved conducting interviews, distributing questionnaires, performing examinations, and analyzing biological samples in the laboratory. Comprehensive information about NHANES can be found online. The National Center for Health Statistics institutional review board approved the NHANES study protocol. Written informed consent was obtained from all NHANES participants. Our analysis did not require additional approval from the Institutional Research Board (IRB). The data used in this study is publicly accessible.^1^ Figure 1 shows the flow chart of study participants.

### Endometriosis definition

The definition of endometriosis included any self-reported diagnosis, irrespective of location, severity, or confirmation through laparoscopy. The reproductive health questionnaire assessed prior endometriosis diagnoses in women aged 20–54 by asking, “Has a doctor or other health professional ever told you that you had endometriosis?”

### Serum carotenoid measurements

Following an overnight fast, venous blood from participants was taken using vacutainer tubes with red or royal blue tops, handled by a phlebotomist trained by NHANES (13). To ensure more accurate results, the serum was kept away from sunlight and other forms of full-spectrum radiation (14). Serum concentrations of ɑ-carotene, *β*-carotene, β-cryptoxanthin, lutein/zeaxanthin, and trans-lycopene were measured using high performance liquid chromatography (HPLC) with photodiode array detection (15). The data were expressed in μg/dL. Details on the laboratory procedures for serum carotenoids measurements are available in the NHANES Laboratory/Medical Technologists Procedures Manual (16).

### Covariates

To mitigate the impact of confounding biases, we incorporated potential confounders as covariates in our analysis (17, 18). We took into account covariates including age, race/ethnicity (non-Hispanic White, not non-Hispanic White), education (high school graduate or less, more than high school), family poverty-to-income ratio (PIR) (below poverty [PIR < 1], at or above poverty [PIR ≥ 1]), BMI (calculated as weight in kilograms divided by height in meters squared), cigarette smoking status (ascertained by serum cotinine levels; individuals were classified as a non smoker or not current smoker if cotinine levels were < 3.0 ng/mL and as a current smoker if cotinine levels were ≥ 3.0 ng/mL), age at menarche (<12 years old, ≥12 years old), and oral contraceptive use (ever, never). The confounding factors are shown in Supplementary Figure S1.

### Statistical analysis

The mean and standard error are used to describe continuous variables, while categorical variables are shown as counts and percentages. Serum levels of ɑ-carotene, *β*-carotene, β-cryptoxanthin, lutein/zeaxanthin, and trans-lycopene were sorted into quartiles, using the lowest quartile as the reference category. Weighted multivariate logistic regression analyses were used to estimate odds ratios (ORs) and 95% confidence intervals (CIs) for the relationship between each carotenoid and endometriosis risk. We constructed three models. Model 1 was an unadjusted model that did not include any covariates. Model 2 accounted for age and BMI, whereas Model 3 adjusted for age, BMI, race, education, PIR, cigarette smoking status, age at menarche, and oral contraceptive use. To determine whether ORs for endometriosis changed with increasing quartiles or concentrations of carotenoids, we calculated the *p*-value for trend (assessing dose–response effects). In addition to logistic regression analyses, restricted cubic spline regression with 3 knots at the 10th, 50th and 90th percentiles was carried out to assess potential nonlinearity in the associations between serum levels of carotenoids and endometriosis. Furthermore, we performed subgroup analyses based on age (< 40 years, 40–49 years, 50–54 years), race (non-Hispanic White, not non-Hispanic White), smoking status (non-smokers, active smokers), and oral contraceptive use (ever, never) to investigate whether these factors could influence the association between serum levels of carotenoids and the risk of endometriosis.

Statistical analyses were performed using R software version 4.1.4 (R Foundation for Statistical Computing, Vienna, Austria) and the complex survey design of NHANES was taken into account. Restricted cubic spline regression was done using DataAnalyst.^2^ Throughout the analysis, two-sided tests were utilized, and a *p*-value of less than 0.05 was regarded as indicative of statistical significance.

## Results

Our study included 3,636 participants, with 263 diagnosed with endometriosis. Table 1 shows the participant characteristics based on the presence or absence of endometriosis. Participants with endometriosis were significantly older, with a mean age of 40.3 years, compared to those without endometriosis, who had a mean age of 35.2 years (*p* < 0.001). Additionally, significant differences were observed in race/ethnicity, smoking status, and oral contraceptive use between endometriosis patients and those without the condition (Table 1).

**Table 1:** Characteristics of study participants.

Variable	Total (*n* = 3,636)	Endometriosis (*n* = 263)	Non-endometriosis (*n* = 3,373)	*P*-value
Age, years, mean (SE)	35.6 (10.6)	40.3 (8.4)	35.2 (10.0)	<0.001
Age, *n* (%)^a^				<0.001
<40 years	2,261 (54.0)	113 (37.9)	2,148 (55.7)	
40–49 years	955 (31.8)	105 (47.6)	850 (30.2)	
50–54 years	420 (14.2)	45 (14.5)	375 (14.1)	
Race/ethnicity, *n* (%)^a^				<0.001
Non-Hispanic White	1775 (69.6)	178 (83.0)	1,597 (68.3)	
Not Non-Hispanic White	1861 (30.4)	85 (17.0)	1776 (31.7)	
Education, *n* (%)^a^				0.462
High school or less	1,583 (36.4)	96 (38.7)	1,487 (36.2)	
More than high school	2053 (63.6)	167 (61.3)	1886 (63.8)	
Family income-to-poverty ratio, *n* (%)^a^				0.289
Below poverty	728 (14.7)	37 (12.0)	691 (15.0)	
At or above poverty	2,908 (85.3)	226 (88.0)	2,682 (85.0)	
BMI, kg/m^2^, mean (SE)	28.8 (0.1)	28.6 (0.4)	28.8 (0.1)	0.714
Smoking status, *n* (%)^a^				
Non-smokers	2,754 (75.7)	182 (69.2)	2,572 (76.3)	0.012
Smokers	882 (24.3)	81 (30.8)	801 (23.7)	
Oral contraceptive use, *n* (%)^a^				<0.001
Ever	2,819 (81.1)	239 (91.6)	2,580 (79.9)	
Never	817 (18.9)	24 (8.4)	793 (20.1)	
Age at menarche, years, *n* (%)^a^				0.389
<12 years	873 (22.9)	71 (25.8)	802 (22.6)	
≥12 years	2,763 (77.1)	192 (74.2)	2,571 (77.4)	

SE, standard error; BMI, body mass index.

a Unweighted number and weighted percentage in NHANES.

Table 2 shows the results for analyses on the associations between serum levels of the major carotenoids and endometriosis risk. After controlling for confounding factors (Model 3), we found that serum lutein/zeaxanthin levels were significantly associated with lower risk of endometriosis (Quartile 3 vs. Quartile 1: OR = 0.62, 95% CI: 0.42–0.90; Quartile 4 vs. Quartile 1: OR = 0.54, 95% CI: 0.36–0.81, *P* for trend = 0.001). We did not observe any significant associations of serum levels of ɑ-carotene, *β*-carotene, β-cryptoxanthin, and trans-lycopene with the risk of endometriosis (Table 2). Although serum levels of β-cryptoxanthin appeared to be statistically significantly associated with decreased endometriosis risk in Model 1 (unadjusted) and Model 2 (adjustment for age and BMI), no significant associations were identified in Model 3 when all confounders were taken into account (Table 2).

**Table 2:** Logistic regression analysis for association between the quartile of serum carotenoids, relative to quartile 1, and endometriosis risk.

Carotenoids	Quartile 1	Quartile 2		Quartile 3		Quartile 4		*P* for trend
	OR (95% CI)	OR (95% CI)	*P*	OR (95% CI)	*P*	OR (95% CI)	*P*	
α-carotene								
Range	<1.5	1.5–2.9		2.9–5.6		≥5.6		
Model 1	Reference	1.16 (0.81–1.65)	0.415	1.23 (0.87–1.75)	0.239	0.87 (0.60–1.26)	0.455	0.578
Model 2	Reference	1.12 (0.78–1.61)	0.524	1.06 (0.74–1.52)	0.741	0.69 (0.47–1.02)	0.062	0.065
Model 3	Reference	1.14 (0.79–1.65)	0.474	1.16 (0.80–1.68)	0.444	0.76 (0.50–1.17)	0.212	0.229
β-carotene								
Range	<7.9	7.9–13.0		13.0–23.6		≥23.6		
Model 1	Reference	0.90 (0.63–1.28)	0.545	0.93 (0.65–1.31)	0.667	0.89 (0.62–1.26)	0.499	0.555
Model 2	Reference	0.87 (0.61–1.24)	0.436	0.83 (0.58–1.20)	0.323	0.69 (0.47–1.01)	0.053	0.058
Model 3	Reference	0.89 (0.62–1.28)	0.519	0.94 (0.73–1.53)	0.750	0.77 (0.51–1.15)	0.194	0.244
β-cryptoxanthin								
Range	<5.2	5.2–8.0		8.0–13.4		≥13.4		
Model 1	Reference	0.80 (0.58–1.10)	0.177	0.53 (0.37–0.75)	<0.001	0.46 (0.32–0.67)	<0.001	<0.001
Model 2	Reference	0.82 (0.59–1.13)	0.228	0.55 (0.38–0.79)	0.001	0.46 (0.32–0.68)	<0.001	<0.001
Model 3	Reference	0.97 (0.69–1.37)	0.857	0.78 (0.52–1.19)	0.250	0.83 (0.52–1.33)	0.433	0.527
Lutein/zeaxanthin								
Range	<10.2	10.2–14.2		14.2–19.7		≥19.7		
Model 1	Reference	0.71 (0.51–0.99)	0.042	0.58 (0.41–0.83)	0.003	0.53 (0.37–0.76)	<0.001	<0.001
Model 2	Reference	0.69 (0.49–0.96)	0.027	0.54 (0.38–0.77)	<0.001	0.47 (0.33–0.69)	<0.001	<0.001
Model 3	Reference	0.75 (0.53–1.06)	0.102	0.62 (0.42–0.90)	0.011	0.54 (0.36–0.81)	0.003	0.001
Trans-lycopene								
Range	<15.9	15.9–22.1		22.1–29.4		≥29.4		
Model 1	Reference	0.98 (0.68–1.40)	0.891	0.96 (0.67–1.38)	0.829	1.09 (0.77–1.55)	0.611	0.641
Model 2	Reference	1.05 (0.73–1.51)	0.790	1.08 (0.75–1.55)	0.683	1.23 (0.86–1.76)	0.248	0.253
Model 3	Reference	0.97 (0.67–1.41)	0.892	0.95 (0.66–1.38)	0.800	1.09 (0.75–1.56)	0.657	0.662

OR, odds ratio; CI, confidence interval.

**Figure 2:**
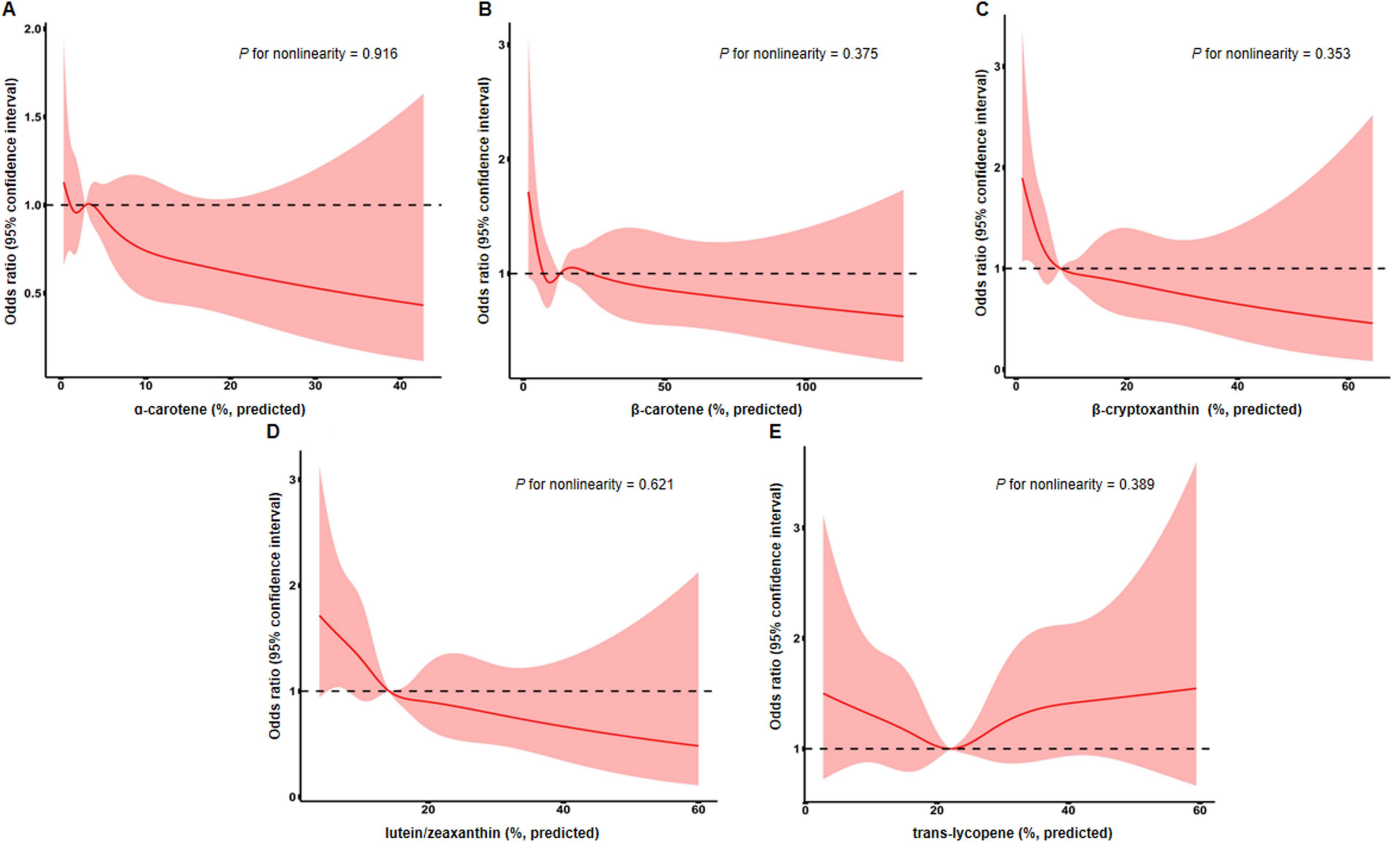
Restricted cubic spline analysis evaluating associations of serum levels of *α*-carotene **(A)**, *β*-carotene **(B)**, β-cryptoxanthin **(C)**, lutein/zeaxanthin **(D)**, and trans-lycopene **(E)** with the risk of endometriosis. Line represents multivariable-adjusted odds ratio, and shaded area represents 95% confidence interval. Models were adjusted for age, BMI, race, education, PIR, cigarette smoking status, age at menarche, and oral contraceptive use.

Next, we conducted restricted cubic spline analyses to evaluate the potential nonlinear relationship between serum levels of these carotenoids and endometriosis, but no statistically significant associations were found (Figure 2).

Furthermore, we carried out subgroup analyses to assess whether the associations of serum levels of these carotenoids with endometriosis would be affected by factors including age, race/ethnicity, smoking status, and oral contraceptive use. We took these factors into account in subgroup analyses in that they showed difference between endometriosis patients and those without the condition (Table 1). The results indicated that serum levels of lutein/zeaxanthin were associated with a reduced risk of endometriosis in individuals under 40 years old, White and non-White populations, smokers, and those with a history of oral contraceptive use (Tables 3–6). Subgroup analyses did not identify any associations of serum levels of ɑ-carotene, *β*-carotene, β-cryptoxanthin, and trans-lycopene with endometriosis (Tables 3–6).

**Table 3:** Association between serum levels of major carotenoids and endometriosis in subgroup analysis according to age.

Quartile (Q1–Q4)	< 40 years (*n* = 2,261)			40–49 years (*n* = 955)			50–54 years (*n* = 420)		
	OR (95% CI)	*P*	*p* for trend	OR (95% CI)	*P*	*P* for trend	OR (95% CI)	*P*	*P* for trend
α-carotene									
Q1	Reference			Reference			Reference		
Q2	1.53 (0.90–2.62)	0.120		0.81 (0.44–1.49)	0.500		1.76 (0.66–4.68)	0.255	
Q3	1.67 (0.94–2.96)	0.078		0.94 (0.52–1.69)	0.840		1.50 (0.58–3.84)	0.402	
Q4	1.36 (0.73–2.54)	0.336	0.930	0.69 (0.36–1.31)	0.250	0.432	0.51 (0.17–1.58)	0.243	0.158
β-carotene									
Q1	Reference			Reference			Reference		
Q2	1.20 (0.71–2.03)	0.494		0.67 (0.36–1.25)	0.213		0.82 (0.34–2.02)	0.673	
Q3	1.13 (0.63–2.03)	0.675		1.04 (0.58–1.84)	0.904		0.61 (0.24–1.61)	0.321	
Q4	1.30 (0.71–2.39)	0.398	0.704	0.73 (0.39–1.38)	0.329	0.657	0.52 (0.21–1.33)	0.172	0.105
β-cryptoxanthin									
Q1	Reference			Reference			Reference		
Q2	0.95 (0.58–1.55)	0.823		0.79 (0.46–1.35)	0.387		1.14 (0.50–2.57)	0.760	
Q3	0.67 (0.38–1.17)	0.157		0.59 (0.32–1.10)	0.096		0.91 (0.36–2.25)	0.832	
Q4	0.68 (0.36–1.27)	0.224	0.082	0.74 (0.39–1.42)	0.369	0.223	0.62 (0.22–1.73)	0.358	0.293
Lutein/zeaxanthin									
Q1	Reference			Reference			Reference		
Q2	0.53 (0.31–0.91)	0.021		0.96 (0.55–1.65)	0.868		1.57 (0.64–3.82)	0.324	
Q3	0.78 (0.47–1.30)	0.342		0.57 (0.30–1.06)	0.076		0.70 (0.24–2.03)	0.512	
Q4	0.40 (0.21–0.75)	0.004	0.002	0.72 (0.38–1.35)	0.310	0.174	1.19 (0.45–3.12)	0.725	0.761
Trans-lycopene									
Q1	Reference			Reference			Reference		
Q2	0.90 (0.49–1.63)	0.728		1.01 (0.57–1.80)	0.974		1.08 (0.48–2.45)	0.851	
Q3	0.70 (0.38–1.29)	0.251		1.60 (0.91–2.80)	0.100		0.55 (0.21–1.46)	0.231	
Q4	1.40 (0.82–2.39)	0.217	0.108	0.72 (0.37–1.39)	0.330	0.822	0.75 (0.31–1.81)	0.525	0.335

OR, odds ratio; CI, confidence interval.

**Table 4:** Association between serum levels of major carotenoids and endometriosis in subgroup analysis according to ethnicity/race.

Quartile (Q1–Q4)	Non-Hispanic White (*n* = 1775)			Not Non-Hispanic White (*n* = 1861)		
	OR (95% CI)	*P*	*P* for trend	OR (95% CI)	*P*	*P* for trend
α-carotene						
Q1	Reference			Reference		
Q2	1.35 (0.86–2.12)	0.190		1.04 (0.54–1.98)	0.915	
Q3	1.57 (0.99–2.50)	0.054		0.90 (0.47–1.72)	0.748	
Q4	1.10 (0.66–1.85)	0.710	0.976	0.61 (0.30–1.27)	0.189	0.129
β-carotene						
Q1	Reference			Reference		
Q2	0.89 (0.57–1.39)	0.604		0.98 (0.52–1.85)	0.945	
Q3	1.13 (0.71–1.80)	0.606		0.76 (0.40–1.44)	0.398	
Q4	1.07 (0.65–1.74)	0.792	0.919	0.58 (0.29–1.17)	0.130	0.066
β-cryptoxanthin						
Q1	Reference			Reference		
Q2	0.97 (0.66–1.44)	0.896		0.76 (0.40–1.47)	0.415	
Q3	0.72 (0.45–1.15)	0.167		0.59 (0.30–1.15)	0.119	
Q4	0.85 (0.49–1.46)	0.560	0.216	0.55 (0.28–1.09)	0.087	0.054
Lutein/zeaxanthin						
Q1	Reference			Reference		
Q2	0.98 (0.65–1.47)	0.927		0.49 (0.25–0.95)	0.034	
Q3	0.84 (0.53–1.31)	0.441		0.40 (0.20–0.78)	0.008	
Q4	0.58 (0.34–0.97)	0.041	0.033	0.53 (0.28–1.01)	0.053	0.045
Trans-lycopene						
Q1	Reference			Reference		
Q2	0.90 (0.57–1.41)	0.637		1.24 (0.65–2.34)	0.514	
Q3	0.76 (0.48–1.20)	0.235		1.45 (0.77–2.72)	0.254	
Q4	1.05 (0.68–1.64)	0.813	0.887	1.04 (0.54–2.00)	0.907	0.771

OR, odds ratio; CI, confidence interval.

**Table 5:** Association between serum levels of major carotenoids and endometriosis in subgroup analysis according to oral contraceptive use.

Quartile (Q1–Q4)	Ever (*n* = 2,819)			Never (*n* = 817)		
	OR (95% CI)	*P*	*P* for trend	OR (95% CI)	*P*	*P* for trend
α-carotene						
Q1	Reference			Reference		
Q2	1.26 (0.86–1.86)	0.246		0.56 (0.16–1.96)	0.364	
Q3	1.22 (0.81–1.82)	0.337		1.01 (0.34–3.04)	0.985	
Q4	0.88 (0.56–1.37)	0.560	0.497	0.27 (0.06–1.21)	0.089	0.192
β-carotene						
Q1	Reference			Reference		
Q2	0.87 (0.59–1.27)	0.460		1.22 (0.37–4.00)	0.747	
Q3	0.93 (0.63–1.38)	0.711		1.32 (0.40–4.37)	0.645	
Q4	0.81 (0.53–1.23)	0.318	0.385	0.56 (0.13–2.33)	0.423	0.498
β-cryptoxanthin						
Q1	Reference			Reference		
Q2	0.88 (0.62–1.25)	0.485		0.90 (0.27–3.02)	0.862	
Q3	0.60 (0.40–1.14)	0.251		1.27 (0.42–3.89)	0.674	
Q4	0.70 (0.45–1.08)	0.104	0.278	0.40 (0.09–1.73)	0.218	0.372
Lutein/zeaxanthin						
Q1	Reference			Reference		
Q2	0.78 (0.54–1.12)	0.176		0.55 (0.18–1.65)	0.287	
Q3	0.67 (0.45–0.99)	0.044		0.21 (0.04–1.02)	0.053	
Q4	0.54 (0.35–0.82)	0.004	0.003	0.61 (0.18–2.06)	0.429	0.257
Trans-lycopene						
Q1	Reference			Reference		
Q2	0.91 (0.62–1.35)	0.654		1.93 (0.63–5.89)	0.248	
Q3	0.92 (0.62–1.35)	0.658		1.43 (0.42–4.84)	0.561	
Q4	1.04 (0.71–1.52)	0.858	0.796	1.70 (0.50–5.77)	0.396	0.455

OR, odds ratio; CI, confidence interval.

**Table 6:** Association between serum levels of major carotenoids and endometriosis in subgroup analysis according to smoking status.

Quartile (Q1–Q4)	Non-smokers (*n* = 2,754)			Smokers (*n* = 882)		
	OR (95% CI)	*P*	*P* for trend	OR (95% CI)	*P*	*P* for trend
α-carotene						
Q1	Reference			Reference		
Q2	1.22 (0.74–1.99)	0.436		1.11 (0.63–1.98)	0.705	
Q3	1.18 (0.72–1.92)	0.508		1.37 (0.74–2.55)	0.317	
Q4	0.88 (0.52–1.46)	0.611	0.421	0.42 (0.14–1.27)	0.124	0.578
β-carotene						
Q1	Reference			Reference		
Q2	0.84 (0.53–1.34)	0.468		0.97 (0.54–1.72)	0.907	
Q3	0.87 (0.55–1.37)	0.538		1.18 (0.61–2.31)	0.622	
Q4	0.69 (0.43–1.11)	0.129	0.154	1.12 (0.51–1.48)	0.711	0.676
β-cryptoxanthin						
Q1	Reference			Reference		
Q2	0.99 (0.64–1.52)	0.947		0.78 (0.45–1.35)	0.378	
Q3	0.71 (0.45–1.13)	0.147		0.64 (0.31–1.31)	0.222	
Q4	0.76 (0.48–1.22)	0.257	0.122	0.15 (0.02–1.11)	0.063	0.323
Lutein/zeaxanthin						
Q1	Reference			Reference		
Q2	0.94 (0.61–1.46)	0.794		0.57 (0.31–1.03)	0.061	
Q3	0.67 (0.42–1.07)	0.090		0.68 (0.35–1.32)	0.249	
Q4	0.68 (0.43–1.09)	0.107	0.058	0.21 (0.06–0.73)	0.014	0.006
Trans-lycopene						
Q1	Reference			Reference		
Q2	1.04 (0.66–1.63)	0.867		0.95 (0.49–1.86)	0.889	
Q3	1.03 (0.66–1.62)	0.891		0.83 (0.42–1.65)	0.589	
Q4	0.94 (0.60–1.49)	0.806	0.808	1.65 (0.88–3.10)	0.117	0.229

OR, odds ratio; CI, confidence interval.

## Discussion

In this population-based study, we found that higher serum levels of lutein/zeaxanthin were associated with a reduced risk of endometriosis. This protective effect was significant only in individuals under 40 years of age, in both White and non-White populations, among smokers, and in those who had ever used oral contraceptives. No non-linear associations were observed between serum lutein/zeaxanthin levels and endometriosis risk. Furthermore, no associations were found between serum levels of ɑ-carotene, β-carotene, β-cryptoxanthin, or trans-lycopene and the risk of endometriosis.

When comparing our results with previous studies, it is important to highlight the differences in study design and measurement methods. While some studies have examined the relationship between dietary carotenoid intake and endometriosis risk (10, 11), our study is one of the few to assess circulating carotenoid levels as a more direct measure of exposure. This distinction is crucial because circulating carotenoids reflect actual bioavailability and metabolic processing, rather than relying on self-reported dietary intake, which is subject to recall bias and inaccuracies. Our findings align with those of Harris et al. (11), who found a reduced risk of endometriosis associated with beta-cryptoxanthin intake, although our study did not identify a significant relationship between beta-cryptoxanthin and endometriosis risk. Our findings are also consistent with those of studies showing that lutein and zeaxanthin played a protective role against other female-related diseases, such as benign breast disease and breast cancer (19, 20). Conversely, Trabert et al. (10) reported an increased risk of endometriosis associated with higher *β*-carotene intake. These contrasting results may reflect the variability in carotenoid absorption, metabolism, and bioactivity across individuals, as well as differences in study populations, methodologies, and sample sizes. Our study, by using serum carotenoid measurements, overcomes some of these limitations and provides a more accurate reflection of carotenoid status in the body.

The inverse association between serum lutein/zeaxanthin levels and endometriosis risk may be attributed to several mechanisms. First, lutein and zeaxanthin are potent antioxidants, and their elevated serum levels may help to mitigate oxidative stress, which is known to play a crucial role in the pathogenesis and progression of endometriosis (21, 22). Elevated oxidative stress markers such as HSP70b’ and malondialdehyde, have been observed in patients with endometriosis compared to control groups (23–25), which can promote inflammation and lesion growth. By neutralizing ROS, lutein and zeaxanthin may reduce oxidative damage and inflammatory signaling, thereby lowering the risk of lesion formation and progression. Second, lutein and zeaxanthin are known to have anti-inflammatory effects. Chronic inflammation is a hallmark of endometriosis, driven by elevated cytokine levels and immune cell infiltration in the pelvic cavity (26). These carotenoids can downregulate the expression of pro-inflammatory cytokines such as IL-6 and IL-1β (27, 28), which are heavily implicated in the inflammatory milieu of endometriosis. Their modulation of immune responses may dampen the chronic inflammatory state, reducing tissue invasion and adhesion formation. Third, lutein and zeaxanthin may influence estrogen metabolism (29, 30). As endometriosis is an estrogen-dependent condition, carotenoids have been shown to modulate estrogen receptor activity (31). By impacting the estrogenic environment, lutein and zeaxanthin may limit the estrogen-driven proliferation of endometrial cells, thus inhibiting the growth of endometriotic lesions.

Our study assessed factors that may modify the relationship between serum carotenoid levels and endometriosis risk. Subgroup analyses suggested that elevated serum levels of lutein and zeaxanthin may offer a protective effect against the development of endometriosis, particularly in individuals under 40 years of age. One possible explanation is that younger women with endometriosis may experience higher levels of oxidative stress and inflammation (21), making them more sensitive to the antioxidative and anti-inflammatory properties of lutein and zeaxanthin. Furthermore, the role of lutein and zeaxanthin in modulating estrogenic pathways might be more pronounced in younger women, given the hormone-driven nature of endometriosis (1). Future studies focusing on age-specific mechanisms of these carotenoids could help clarify the observed age-related effects in the present study. The protective association was consistent across both White and non-White populations, highlighting the potential for broad applicability across different ethnic groups. Moreover, the effect was particularly pronounced in active smokers and participants who had ever used oral contraceptives, suggesting that these subgroups may experience unique oxidative stressors or hormonal influences that amplify the benefit of lutein and zeaxanthin’s antioxidative properties. These carotenoids are known to mitigate oxidative stress and inflammation, both of which play central roles in the pathophysiology of endometriosis. Therefore, modulation of oxidative pathways and inflammation by lutein and zeaxanthin may represent a key mechanism in reducing the risk of disease onset, particularly in higher-risk populations, such as women of reproductive age (1), smokers (32, 33), and those with specific hormonal exposures (34).

In terms of clinical implications, our findings raise the possibility that dietary recommendations or supplementation with lutein/zeaxanthin-rich foods could serve as a complementary approach to reduce the risk of endometriosis. Foods such as dark leafy greens, corn, eggs, and certain fruits are rich in lutein and zeaxanthin. However, while our results are promising, it is important to note that dietary interventions should be considered as part of a broader strategy that includes medical management, particularly since endometriosis is a multifactorial condition involving both genetic and environmental factors (1). Further clinical trials are needed to assess whether increasing lutein/zeaxanthin intake can indeed reduce the incidence or severity of endometriosis.

Our study has several limitations that should be considered when interpreting the findings. First, while we conducted a cross-sectional analysis on the data from the NHANES 2001–2006 cycles, the study’s design limited the ability to establish a temporal relationship between serum carotenoid levels and endometriosis risk, preventing causal inferences. Second, endometriosis diagnosis in NHANES is self-reported, which may introduce recall bias and lead to potential misclassification of cases. Additionally, the serum carotenoid levels were measured at a single point in time, failing to account for potential fluctuations over the course of the disease. Finally, unmeasured confounders, such as environmental exposures and genetic predispositions, could have influenced both carotenoid levels and endometriosis risk, and while we adjusted for several known covariates, residual confounding may still be present.

## Conclusion

In summary, this study found an inverse association between serum lutein/zeaxanthin levels and the risk of endometriosis, with a particularly pronounced protective effect in individuals under 40 years of age, as well as in both White and non-White populations, smokers, and those who had ever used oral contraceptives. However, no significant associations were found between serum levels of ɑ-carotene, *β*-carotene, β-cryptoxanthin, or trans-lycopene and endometriosis risk. Future research with a prospective design and a focus on establishing causality is warranted to validate these findings.

## Data Availability

Publicly available datasets were analyzed in this study. This data can be found here: https://wwwn.cdc.gov/nchs/nhanes/.

## Footnotes

^1^ https://wwwn.cdc.gov/nchs/nhanes/

^2^ https://medata-ai.github.io/DataAnalyst

## Supplementary material

SUPPLEMENTARY FIGURE S1 Confounding factors taken into account in the analyses.

## References

[1] Horne AW Missmer SA. Pathophysiology, diagnosis, and management of endometriosis. BMJ. (2022) 379:e070750. doi: 10.1136/bmj-2022-070750 36375827

[2] Saunders PTK Whitaker LHR Horne AW. Endometriosis: improvements and challenges in diagnosis and symptom management. Cell Rep Med. (2024) 5:101596. doi: 10.1016/j.xcrm.2024.101596 38897171 PMC11228648

[3] Sarria-Santamera A Orazumbekova B Terzic M Issanov A Chaowen C Asúnsolo-Del-Barco A. Systematic review and Meta-analysis of incidence and prevalence of endometriosis. Healthcare (Basel). (2020) 9:29. doi: 10.3390/healthcare9010029 33396813 PMC7824417

[4] Osmanlıoğlu Ş Sanlier N. The relationship between endometriosis and diet. Hum Fertil (Camb). (2023) 26:649–64. doi: 10.1080/14647273.2021.1995900 34706611

[5] Barnard ND Holtz DN Schmidt N Kolipaka S Hata E Sutton M. Nutrition in the prevention and treatment of endometriosis: a review. Front Nutr. (2023) 10:1089891. doi: 10.3389/fnut.2023.1089891 36875844 PMC9983692

[6] Bohn T Balbuena E Ulus H Iddir M Wang G Crook N. Carotenoids in health as studied by omics-related endpoints. Adv Nutr. (2023) 14:1538–78. doi: 10.1016/j.advnut.2023.09.002 37678712 PMC10721521

[7] Mueller L Boehm V. Antioxidant activity of β-carotene compounds in different in vitro assays. Molecules. (2011) 16:1055–69. doi: 10.3390/molecules16021055 21350393 PMC6259600

[8] Saini RK Prasad P Lokesh V Shang X Shin J Keum YS. Carotenoids: dietary sources, extraction, encapsulation, bioavailability, and health benefits-a review of recent advancements. Antioxidants (Basel). (2022) 11:795. doi: 10.3390/antiox11040795 35453480 PMC9025559

[9] Udensi J Loughman J Loskutova E Byrne HJ. Raman spectroscopy of carotenoid compounds for clinical applications-a review. Molecules. (2022) 27:9017. doi: 10.3390/molecules27249017 36558154 PMC9784873

[10] Trabert B Peters U De Roos AJ Scholes D Holt VL. Diet and risk of endometriosis in a population-based case-control study. Br J Nutr. (2011) 105:459–67. doi: 10.1017/S0007114510003661 20875189 PMC3374872

[11] Harris HR Eke AC Chavarro JE Missmer SA. Fruit and vegetable consumption and risk of endometriosis. Hum Reprod. (2018) 33:715–27. doi: 10.1093/humrep/dey014 29401293 PMC6018917

[12] National Center for Health Statistics. NHANES survey methods and analytic guidelines. Available at: https://wwwn.cdc.gov/nchs/nhanes/analyticguidelines.aspx#sample-design (Accessed September 14, 2024).

[13] Zhang C Li K Xu SN Zhang JK Ma MH Liu Y. Higher serum carotenoid concentrations were associated with the lower risk of cancer-related death: evidence from the National Health and nutrition examination survey. Nutr Res. (2024) 126:88–98. doi: 10.1016/j.nutres.2024.03.012 38642420

[14] Bruno RR Rosa FC Nahas PC de Branco FMS de Oliveira EP. Serum ɑ-carotene, but not other antioxidants, is positively associated with muscle strength in older adults: NHANES 2001–2002. Antioxidants (Basel). (2022) 11:2386. doi: 10.3390/antiox11122386 36552594 PMC9774096

[15] Beydoun MA Canas JA Beydoun HA Chen X Shroff MR Zonderman AB. Serum antioxidant concentrations and metabolic syndrome are associated among U.S. adolescents in recent national surveys. J Nutr. (2012) 142:1693–704. doi: 10.3945/jn.112.160416 22810988 PMC3417831

[16] Mazidi M Kengne AP Katsiki N Mikhailidis DP Banach M. Inverse association between serum antioxidant levels and inflammatory markers is moderated by adiposity: a report based on a large representative population sample of American adults. Br J Nutr. (2018) 120:1272–8. doi: 10.1017/S0007114518002581 30378506

[17] Han GM Meza JL Soliman GA Islam KM Watanabe-Galloway S. Higher levels of serum lycopene are associated with reduced mortality in individuals with metabolic syndrome. Nutr Res. (2016) 36:402–7. doi: 10.1016/j.nutres.2016.01.003 27101758

[18] Lee AW Eata V. Association of environmental phenols with endometriosis and uterine leiomyoma: an analysis of NHANES, 2003–2006. Reprod Toxicol. (2022) 113:30–4. doi: 10.1016/j.reprotox.2022.08.003 35948171

[19] Boeke CE Tamimi RM Berkey CS Colditz GA Eliassen AH Malspeis S. Adolescent carotenoid intake and benign breast disease. Pediatrics. (2014) 133:e1292–8. doi: 10.1542/peds.2013-3844 24709924 PMC4006443

[20] Park SH Lee J Jung SY Park S Kang YH Kim J. Association between dietary carotenoid intake and breast cancer risk: a case-control study among Korean women. Int J Food Sci Nutr. (2024) 75:496–508. doi: 10.1080/09637486.2024.2358111 38828549

[21] Ansariniya H Yavari A Javaheri A Zare F. Oxidative stress-related effects on various aspects of endometriosis. Am J Reprod Immunol. (2022) 88:e13593. doi: 10.1111/aji.13593 35781369

[22] Clower L Fleshman T Geldenhuys WJ Santanam N. Targeting oxidative stress involved in endometriosis and its pain. Biomol Ther. (2022) 12:1055. doi: 10.3390/biom12081055 PMC9405905 36008949

[23] Lambrinoudaki IV Augoulea A Christodoulakos GE Economou EV Kaparos G Kontoravdis A. Measurable serum markers of oxidative stress response in women with endometriosis. Fertil Steril. (2009) 91:46–50. doi: 10.1016/j.fertnstert.2007.11.021 18206876

[24] Matsuzaki S Schubert B. Oxidative stress status in normal ovarian cortex surrounding ovarian endometriosis. Fertil Steril. (2010) 93:2431–2. doi: 10.1016/j.fertnstert.2009.08.068 19819438

[25] Nasiri N Moini A Eftekhari-Yazdi P Karimian L Salman-Yazdi R Arabipoor A. Oxidative stress statues in serum and follicular fluid of women with endometriosis. Cell J. (2017) 18:582–7. doi: 10.22074/cellj.2016.4724 28042542 PMC5086336

[26] Symons LK Miller JE Kay VR Marks RM Liblik K Koti M. The Immunopathophysiology of endometriosis. Trends Mol Med. (2018) 24:748–62. doi: 10.1016/j.molmed.2018.07.004 30054239

[27] Chung RWS Leanderson P Lundberg AK Jonasson L. Lutein exerts anti-inflammatory effects in patients with coronary artery disease. Atherosclerosis. (2017) 262:87–93. doi: 10.1016/j.atherosclerosis.2017.05.008 28527371

[28] Stringham NT Holmes PV Stringham JM. Effects of macular xanthophyll supplementation on brain-derived neurotrophic factor, pro-inflammatory cytokines, and cognitive performance. Physiol Behav. (2019) 211:112650. doi: 10.1016/j.physbeh.2019.112650 31425700

[29] Maggio M de Vita F Lauretani F Bandinelli S Semba RD Bartali B. Relationship between carotenoids, retinol, and estradiol levels in older women. Nutrients. (2015) 7:6506–19. doi: 10.3390/nu7085296 26251919 PMC4555135

[30] Tamimi RM Hankinson SE Campos H Spiegelman D Zhang S Colditz GA. Plasma carotenoids, retinol, and tocopherols and risk of breast cancer. Am J Epidemiol. (2005) 161:153–60. doi: 10.1093/aje/kwi030 15632265

[31] Hirsch K Atzmon A Danilenko M Levy J Sharoni Y. Lycopene and other carotenoids inhibit estrogenic activity of 17beta-estradiol and genistein in cancer cells. Breast Cancer Res Treat. (2007) 104:221–30. doi: 10.1007/s10549-006-9405-7 17051425

[32] Andolf E Thorsell M Källén K. Caesarean section and risk for endometriosis: a prospective cohort study of Swedish registries. BJOG. (2013) 120:1061–5. doi: 10.1111/1471-0528.12236 23663181

[33] Yen CF Kim MR Lee CL. Epidemiologic factors associated with endometriosis in East Asia. Gynecol Minim Invasive Ther. (2019) 8:4–11. doi: 10.4103/GMIT.GMIT_83_18 30783582 PMC6367920

[34] Capezzuoli T Rossi M La Torre F Vannuccini S Petraglia F. Hormonal drugs for the treatment of endometriosis. Curr Opin Pharmacol. (2022) 67:102311. doi: 10.1016/j.coph.2022.102311 36279764

