# Supplementary figures and images for: Association between serum carotenoids levels and endometriosis risk: evidence from the National Health and Nutrition Examination Survey
Source: Front Nutr. 2025 Feb 4;12:1513191. doi: 10.3389/fnut.2025.1513191 (PMC11832354; doi:10.3389/fnut.2025.1513191)

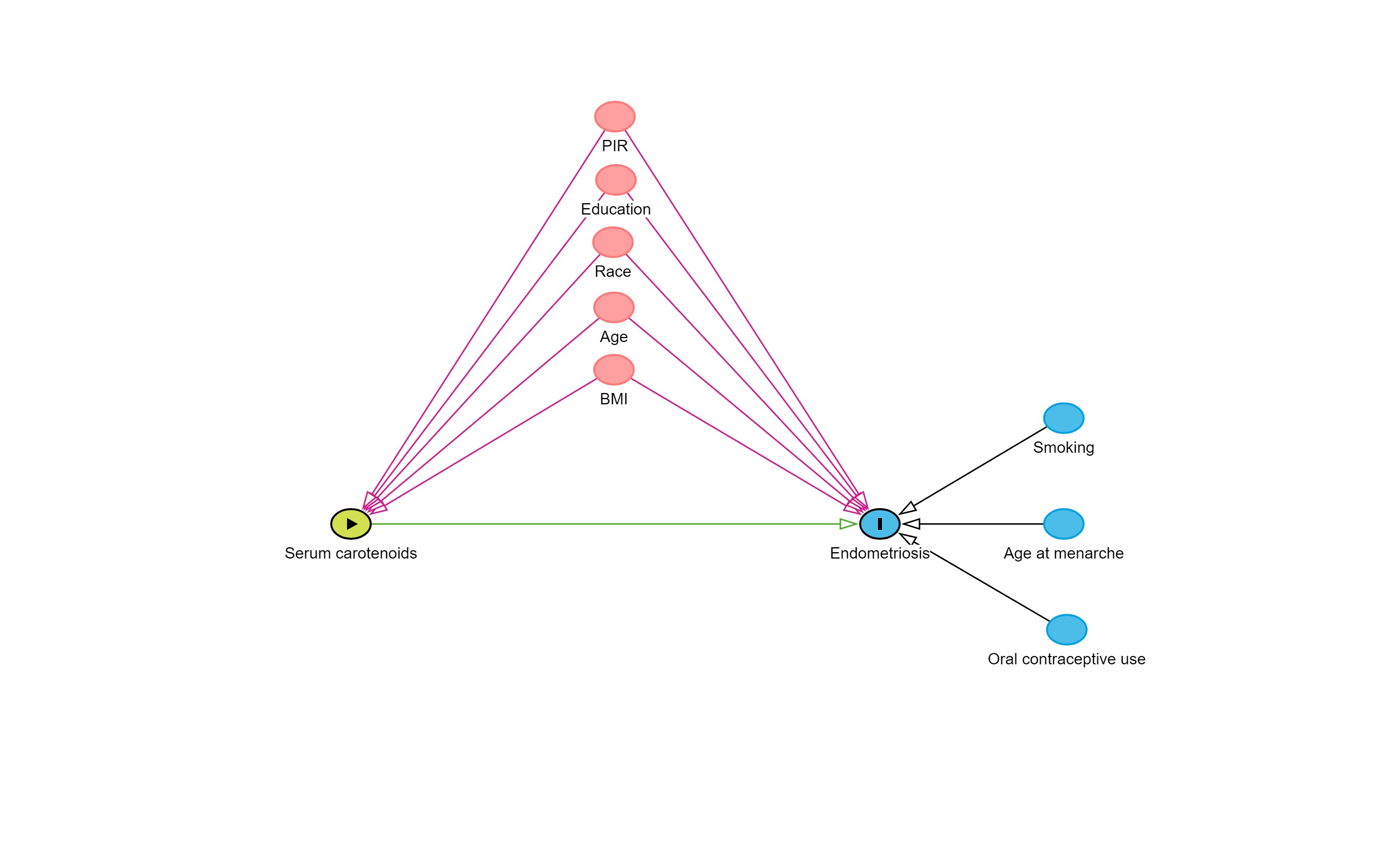

Supplement: SUPPLEMENTARY FIGURE S1 — Confounding factors taken into account in the analyses. [file Image_1.jpeg]
